# Talin-1 inhibits Smurf1-mediated Stat3 degradation to modulate β-cell proliferation and mass in mice

**DOI:** 10.1038/s41419-023-06235-8

**Published:** 2023-10-31

**Authors:** Xiaoting Hou, Yangshan Chen, Bo Zhou, Wanze Tang, Zhen Ding, Litong Chen, Yun Wu, Hongyu Yang, Changzheng Du, Dazhi Yang, Guixing Ma, Huiling Cao

**Affiliations:** 1 Department of Biochemistry, School of Medicine, Southern University of Science and Technology, Key University Laboratory of Metabolism and Health of Guangdong, Guangdong Provincial Key Laboratory of Cell Microenvironment and Disease Research, Southern University of Science and Technology, Shenzhen, 518055 China; 2 The First Affiliated Hospital, Southern University of Science and Technology, Shenzhen, 518055 China; 3 Department of Oral and Maxillofacial Surgery, Stomatological Center, Peking University Shenzhen Hospital; Guangdong Provincial High-level Clinical Key Specialty; Guangdong Province Engineering Research Center of Oral Disease Diagnosis and Treatment; The Institute of Stomatology, Peking University Shenzhen Hospital, Shenzhen Peking University; The Hong Kong University of Science and Technology Medical Center, Guangdong, China

**Keywords:** Type 1 diabetes, Mechanisms of disease

## Abstract

Insufficient pancreatic β-cell mass and reduced insulin expression are key events in the pathogenesis of diabetes mellitus (DM). Here we demonstrate the high expression of Talin-1 in β-cells and that deficiency of Talin-1 reduces β-cell proliferation, which leads to reduced β-cell mass and insulin expression, thus causing glucose intolerance without affecting peripheral insulin sensitivity in mice. High-fat diet fed exerbates these phenotypes. Mechanistically, Talin-1 interacts with the E3 ligase smad ubiquitination regulatory factor 1 (Smurf1), which prohibits ubiquitination of the signal transducer and activator of transcription 3 (Stat3) mediated by Smurf1, and ablation of Talin-1 enhances Smurf1-mediated ubiquitination of Stat3, leading to decreased β-cell proliferation and mass. Furthermore, haploinsufficiency of *Talin-1* and *Stat3* genes, but not that of either gene, in β-cell in mice significantly impairs glucose tolerance and insulin expression, indicating that both factors indeed function in the same genetic pathway. Finally, inducible deletion Talin-1 in β-cell causes glucose intolerance in adult mice. Collectively, our findings reveal that Talin-1 functions as a crucial regulator of β-cell mass, and highlight its potential as a therapeutic target for DM patients.

## Introduction

Diabetes mellitus (DM) is a chronic metabolic disorder characterized by impaired insulin secretion or insulin resistance, which affects approximately 10.5% of the global adult population aged between 20–79 years [1, 2]. Type 1 diabetes (T1D) and type 2 diabetes (T2D) are two distinct types of diabetes mellitus, both of which are characterized by the dysfunction and reduction of pancreatic islet β-cells [3, 4]. This dysfunction is a result of dysregulated β-cell proliferation, differentiation, and apoptosis. In T1D, insulin dependence is a defining feature, while in T2D, β-cell dysfunction and loss occur as a result of various events including insulin resistance and metabolic dysfunction [5–8].

Since insulin, the sole hypoglycemic hormone, is synthesized by β-cells, understanding the underlying mechanisms that regulate the preservation of β-cell mass is crucial for maintaining glucose homeostasis. A decline in β-cell mass results in insulin insufficiency which contributes to the onset of both T1D and T2D, and thus preserving β-cell mass is a potential therapeutic approach for the treatment of DM [9–12]. Stat3 is known as a key transcription factor that promotes cell growth and proliferation through controlling the expression of Cyclin D1, Cyclin B1, c-Myc, etc. [13–18]. Stat3 deficiency in Pdx1-expressing cells hinders β-cell regeneration through reducing Cyclin D1 expression [19]. While the importance of Stat3 in regulating β-cell proliferation is well characterized, mechanisms controlling Stat3 expression and activity in β-cell are incompletely understood.

During the course of pancreatic development and the advancement of both T1D and T2D, β-cells are encompassed by a capsule composed of capillary extracellular matrix (ECM). This capsule undergoes alterations in its composition and organization that facilitate cell migration, intracellular biochemical signaling transduction, modulation of downstream signaling pathways, and modulation of cell fate [20–23]. Focal adhesion (FA) proteins play key roles in mediating integrins related signaling pathways and modulate many important cellular processes, including cell-ECM interaction, cell proliferation and survival, mechanosensation and signal transduction [24–27]. Studies have demonstrated that integrin and key FA proteins, including Kindlin-2, Pinch, etc., play critical roles in maintaining glucose homeostasis through their expression in β-cells or other tissues [27–31]. Talin, one of the key FA proteins, is known to be an essential regulator of integrin-mediated signaling [32–36]. In mammals, Talin family consists of Talin-1 and Talin-2, encoded by *Tln1* and *Tln2*, respectively. Of the two isoforms, *Tln1* is more ubiquitously expressed than *Tln2* throughout the body. Nicholas H. Brown *et al*. and Amanda Haage et al. reported that knockout Talin-1 in *drosophila* and mice universally lead to defects in integrin-mediated adhesion [37, 38]. Zhang et al. showed KN motif and ankyrin repeat domain-containing proteins 4 (KANK4) can link vascular endothelial growth factor receptor 2 (VEGFR2) to TALIN-1, resulting in enhanced activation of VEGFR2 and increased proliferation of endothelial cells (ECs) [39]. Both Talin-1 expression levels and mutation have been associated with tumor progression and metastasis across several types of cancer, including hepatocellular carcinoma, breast cancer, and prostate cancer [40–43]. Moreover, absence of Talin-1 in cardiac fibroblasts (CFs) would result in augmented ventricular cardiomyocyte hypertrophy in response to pressure overload [44]. Talin-1 can also regulate platelet and leukocyte functions and promote angiogenesis in mice [45, 46]. However, the role of Talin-1 in β-cell in maintaining glucose homeostasis remains unknown.

In this study, we generate β-cell specific *Talin-1* knockout mice to investigate the role of Talin-1 in regulation of β-cell differentiation and function. After comprehensive analyses of control and mutant mice, we demonstrate the critical role of Talin-1 in maintaining β-cell mass. Our in vivo and in vitro studies reveal that deficiency of Talin-1 dramatically impairs β-cell proliferation and mass, leading to a drastic reduction in insulin expression and secretion, resulting in impaired glucose tolerance and glucose-stimulated insulin secretion (GSIS). Mechanistically, we find that Smurf1 interacts with and ubiquitinates Stat3, which is blocked by Talin-1 through its interaction with Smurf1. Talin-1 deficiency enhances Stat3 ubiquitination by Smurf1, resulting in downregulation of both p-Stat3 and Stat3 expression. Additionally, mice with haploinsufficiency of *Talin-1* and *Stat3* genes, but not that of either gene, in β-cells exhibit significant glucose intolerance and reduced insulin expression, suggesting that both factors function in the same genetic pathway. Furthermore, inducible ablation of Talin-1 in β-cells of adult mice leads to similar phenotypes with impaired glucose tolerance and GSIS. Collectively, our findings suggest that Talin-1 in β-cells functions as a key regulator of glucose homeostasis by modulating β-cell proliferation and mass through controlling Smurf1-mediated Stat3 ubiquitination and proteasomal degradation.

## Results

### Talin-1 is abundantly expressed in the pancreas and its expression in β-cells is altered under diabetic conditions

**Fig. 1:**
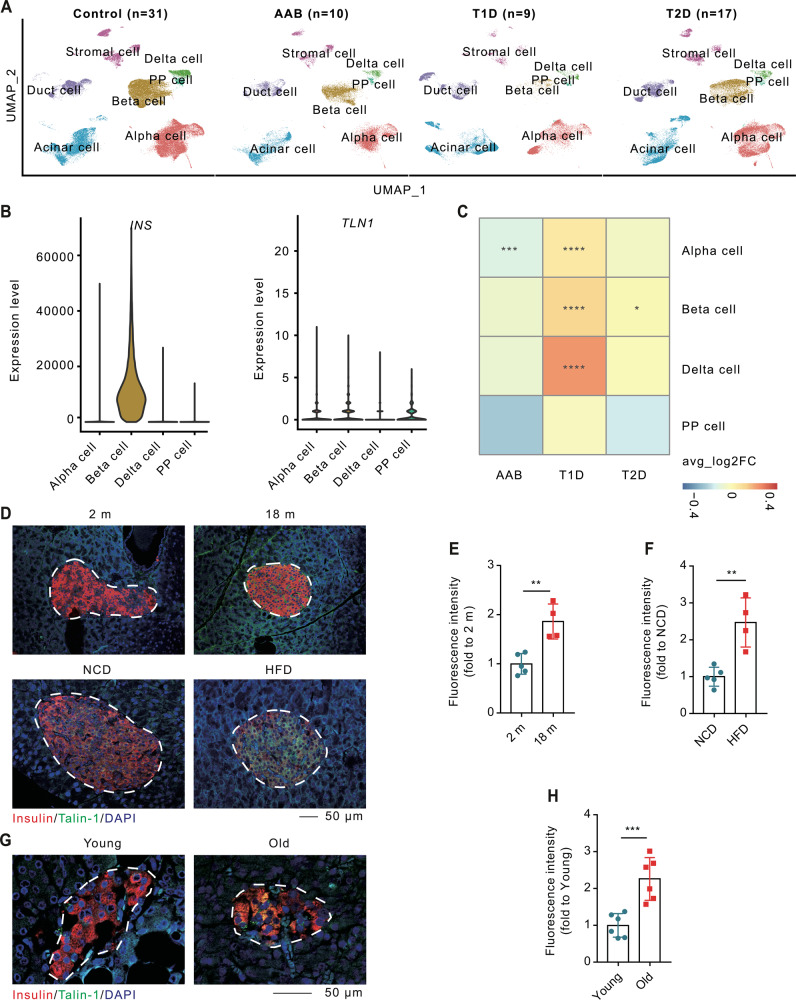
Talin-1 expression is upregulated under diabetic conditions. **A** Expression of marker genes for each cell type in UMAP visualization. **B** Expression of insulin and Talin-1 in pancreatic cells. **C** Expression of Talin-1 in different cell types of islets in patients with Type 1 diabetes (T1D) or Type 2 diabetes (T2D), comparing with healthy control. **D** Immunofluorescence (IF) staining of mouse pancreatic sections with antibodies against Talin-1, insulin and DAPI. Upper: 2-month-old and 18-month-old. *N* = 5 mice for 2-month-old group, *N* = 4 mice for 18-month-old group. Bottom: mice treated with normal chow diet (NCD) and high-fat diet (HFD), *N* = 5 mice for NCD, *N* = 4 mice for HFD. Scale bar, 50 μm. **E**, **F** Talin-1 positive β-cell calculated based on (**D**). **G** IF staining of human pancreatic sections of different age. Scale bar, 50 μm. **H** Talin-1 positive β-cell calculated based on (**G**), *N* = 6 per group. Results are expressed as mean ± standard deviation (s.d.) and Student’s *t* test was performed to analyze the difference between the two groups. *p < 0.05, **p < 0.01, ***p < 0.001, versus control.

To investigate the potential association between *Talin-1* gene expression and diabetes mellitus (DM) in human, we conducted a re-analysis of the single-cell RNA sequencing (scRNA-seq) dataset obtained from human islets in the PANC-DB (Fig. 1A) [47]. Our analysis revealed a broad distribution of Talin-1 expression across the profiled islet cells, suggesting a pivotal role of Talin-1 in human islets (Fig. 1B, Supplementary Fig. 1). Notably, Talin-1 was markedly and significantly up-regulated in β-cells of both T1D and T2D (Fig. 1C). Hence, our scRNA-seq analysis revealed a substantial upregulation of Talin-1 in patients with diabetes.

To verify above results, we conducted immunofluorescent (IF) staining of mouse pancreatic samples to detect signals of Talin-1 and insulin. Consistently, we also observed a prominent upregulation of Talin-1 in islets from aged (18-month-old) or high-fat diet (HFD)-treated mice (Fig. 1D–F). Furthermore, we also detected a higher level of Talin-1 in human pancreatic islet from a 65-year-old patient compared with that of a 32-year-old patient (Fig. 1G, H). Notably, impaired β-cell functions have been reported under conditions of T1D, Type 2 T2D, aging, and HFD-induced metabolic stress [48–51]. Thus, our findings provide compelling evidence that the progression of diatetes is accompanied with increased Talin-1 expression in islets. Nevertheless, the mechanistic link between Talin-1 and β-cell functions in these pathological contexts remains unknown and warrants further investigation.

### Talin-1 deficiency in β-cell causes glucose intolerance and reduced insulin release

**Fig. 2:**
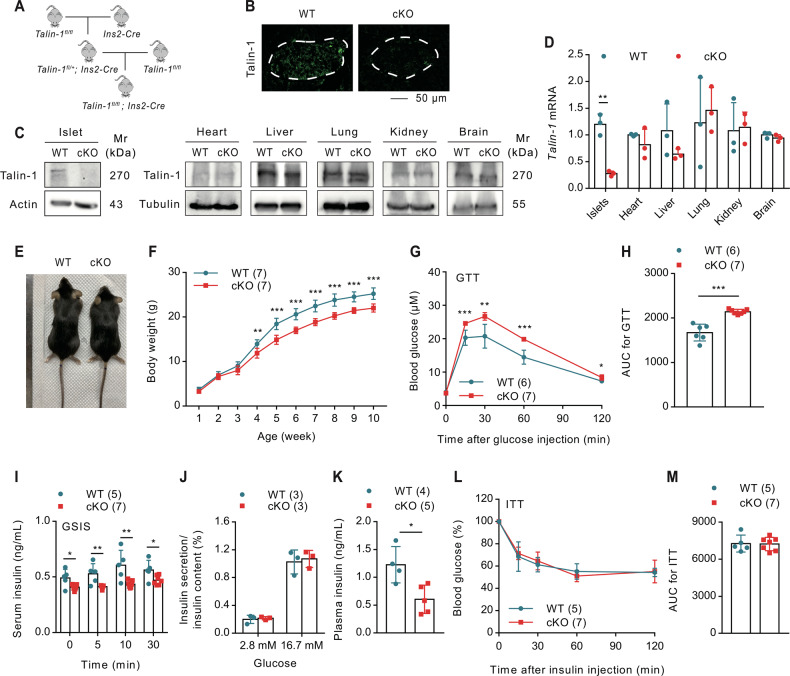
Talin-1 deficiency causes glucose intolerance without affecting insulin sensitivity. **A** Breeding strategy for generating mice with β-cell specific deletion of Talin-1. **B** IF staining of pancreatic sections of 3-month-old male cKO or WT mice with anti-Talin-1 antibody. Scale bar, 50 μm. **C** Western blot analyses. Protein extracts isolated from the indicated tissues of 3-month-old male cKO and WT mice were subjected to western blot analyses for Talin-1 expression. Actin and tubulin were used as a loading control. **D** Quantitative reverse transcriptase-polymerase chain reaction (qPCR) analyses. Total RNAs isolated from the indicated tissues of 3-month-old male cKO and WT mice were subjected to qPCR for *Talin-1* expression. *Tln1* mRNA was normalized to *Gapdh* mRNA. **E** Gross appearance of 3-month-old male cKO and WT mice. **F** Mice growth curve. *N* = 7 mice per group. **G** Glucose tolerance test (GTT). 10-week-old male mice were subjected to GTT assay. Briefly, mice were fasted overnight and then were intraperitoneally injected with glucose (2 g/kg body weight). *N* = 6 mice for WT group and *N* = 7 mice for cKO group. **H** Area under the curve (AUC) calculated based on (**G**). **I** In vivo glucose-stimulated insulin secretion assay (GSIS). 12-week-old male mice were subjected to GSIS. Briefly, mice were fasted overnight and then were intraperitoneally injected with glucose (2 g/kg body weight). Blood samples were collected at 0, 5, 10, 30 min after glucose injection and insulin levels were detected by ELISA assay. *N* = 7 mice for WT group and *N* = 7 mice for cKO group. **J** In vitro GSIS. Islets isolated from 2-month-old male mice were treated with 2.8 or 16.7 mM glucose. Insulin levels in the supernatant and in the total protein extracted from the islets were measured by ELISA, and insulin level in the supernatant was normalized to total insulin content. **K** Fasting blood insulin level. 2-month-old male mice were fasted overnight, and blood insulin level was measured by ELISA assay. *N* = 4 mice for WT group and *N* = 5 mice for cKO group. **L** Insulin tolerance test (ITT). 11- week-old male mice were fasted for 6-7 h and then were intraperitoneally injected with a single dose of recombinant human insulin at 1 U/kg body weight. Blood glucose levels were detected by ELISA assay at 0, 30, 60, 90, and 120 min after insulin injection. *N* = 5 mice for WT group and *N* = 7 mice for cKO group. **M** AUC calculated based on (**L**). Results are expressed as mean ± standard deviation (s.d.) and unpaired two-tailed Student’s *t* test (two groups) or one-way ANOVA (multiple groups) was performed to analyze the difference between the two groups. *p < 0.05, **p < 0.01, ***p < 0.001, versus control.

The above results aroused our interest in studying the role of Talin-1 in the regulation of glucose homeostasis. For this purpose, we generated β-cell specific Talin-1 knockout mice by breeding *Talin-1*^*fl/fl*^ mice with the *Ins2*^*Cre*^ mice, that is the *Talin-1*^*fl/fl*^*; Ins2*^*Cre*^ mice (hereafter referred to as cKO). We used *Talin-1*^*fl/fl*^ mice (hereafter referred to as WT) as control. The mating strategy is presented in Fig. 2A. The cKO mice were born in accordance with Mendel’s laws of heredity. IF staining of pancreatic tissues using antibodies against Talin-1 demonstrated a significant reduction of Talin-1 expression in islet β-cells of cKO mice compared with that of WT mice (Fig. 2B). Furthermore, Western blotting (WB) and quantitative real-time reverse transcriptase-polymerase chain reaction (qPCR) analyses also revealed a substantial decrease in Talin-1 levels in isolated islets of cKO mice compared to WT counterparts, while Talin-1 expression in other organs, including heart, liver, lung, kidney, and brain, remained unchanged (Fig. 2C, D). These findings substantiated the specific and effective knockout of Talin-1 expression in islet β-cells of the cKO mice. Notably, long-term body weight monitoring from birth in mice showed growth retardation of the cKO mice starting at 4 weeks of age (Fig. 2E, F).

We next conducted a glucose tolerance test (GTT) to evaluate the impact of Talin-1 deficiency on glucose homeostasis and insulin secretion in cKO mice. After starving overnight, mice was intraperitoneally (i.p.) administrated of glucose (2 g/kg body weight), and blood samples were collected at 0, 15, 30, 60, and 120 min for measurement of blood glucose. Results showed that 3-month-old male cKO mice exhibited significantly elevated blood glucose levels compared to sex- and age-matched WT littermates at 15, 30, and 60 min (Fig. 2G, H), indicating impaired glucose clearance from the blood of cKO mice.

Furthermore, we performed glucose-stimulated insulin secretion (GSIS) assays to investigate the influence of Talin-1 deficiency on insulin secretion in mice. Blood insulin levels were measured at 0, 5, 15, and 30 min after i.p. injection of glucose (2 g/kg body weight). Insulin secretion was markedly reduced in cKO mice compared to that of the WT mice (Fig. 2I). To further explore the possible causes of reduced insulin release in cKO mice, we isolated primary islets from cKO and WT mice and treated them with 2.8- or 16.7-mM glucose in vitro, then we measured the levels of insulin protein in the supernatant and in the whole cell protein extracts of the islets. Insulin secretion was robustly elevated by about 5-fold in both WT and cKO islets upon exposure to 16.7 mM glucose, indicating that the reduced in vivo insulin secretion in cKO mice was due to reduced insulin expression (Fig. 2J).

Although GTT assays revealed that there was no obvious difference in the level of fasting blood glucose between cKO mice and WT mice, the level of fasting blood insulin in cKO mice was severely reduced by Talin-1 loss in cKO mice (Fig. 2K). Then we performed insulin tolerance tests (ITT) and found that there was no significant differences in insulin sensitivity between cKO mice and WT mice (Fig. 2L, M). Collectively, these results provide compelling evidence that deficiency of Talin-1 in β-cells impairs glucose homeostasis through suppressing insulin expression.

### Ablation of β-cell Talin-1 reduces β-cell proliferation and mass

**Fig. 3:**
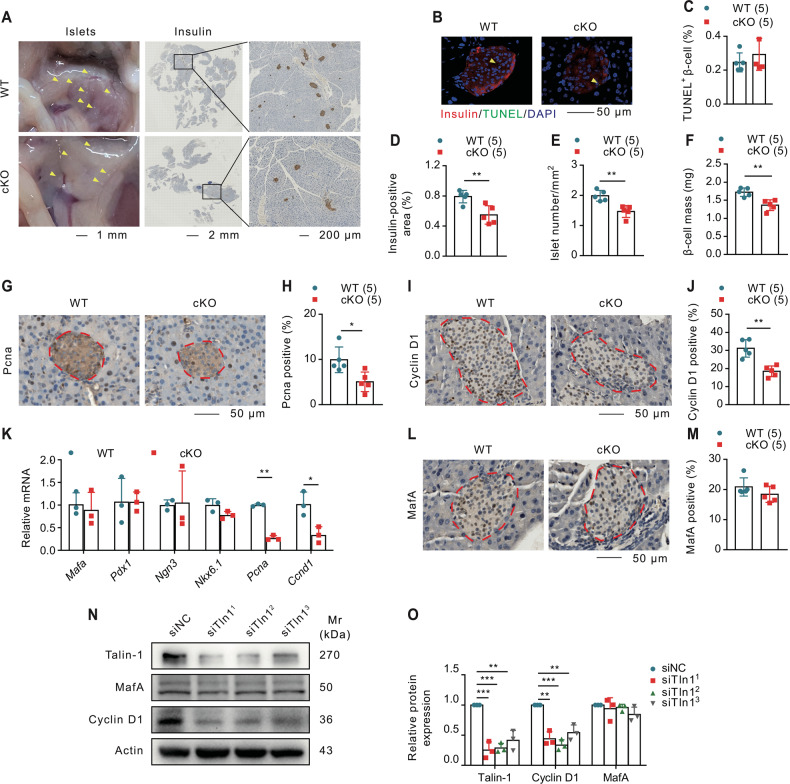
Talin-1 deficiency reduces β-cell proliferation and mass. **A** Pancreas and insulin immunohistochemistry (IHC) staining. Scale bar left, 1 mm. Scale bar middle, 2 mm. Scale bar right, 200 μm. **B**, **C** TUNEL staining of NCD-fed WT and cKO mice. Scale bar, 50 μm. Quantitative data (**C**). **D** Insulin-positive area. **E** Islet number per pancreas cross area. **F** β-cell mass. **G**, **H** IHC staining for Pcna. Scale bar, 50 μm. Quantitative data (**H**). **I**, **J** IHC staining for Cyclin D1. Scale bar, 50 μm. Quantitative data (**J**). *N* = 5 mice per group. **K** QPCR analyses. Total RNAs isolated from the islets of 2-month-old male cKO and WT mice were subjected to qPCR for *Mafa*, *Pdx1*, *Ngn3*, *Nkx6.1*, *Pcna*, and *Ccnd1* expression. All mRNA was normalized to *Gapdh* mRNA. Student’s *t* test was performed using the average values of triplicates from three independent experiments. **L**, **M** IHC staining for MafA. Scale bar, 50 μm. Quantitative data (**M**). *N* = 5 mice per group. **N**, **O** Western blot analyses. INS1 cells co-transfected with the plasmids of siTln1 for 48 h and next the protein extracts were subjected to Western blot analyses with indicated antibodies. Quantitative data from three independent experiments (**O**). Results are expressed as mean ± standard deviation (s.d.) and unpaired two-tailed Student’s *t* test (two groups) or one-way ANOVA (multiple groups) was performed to analyze the difference between the two groups. *p < 0.05, **p < 0.01, ***p < 0.001, versus control.

Numerous studies have implicated increased apoptosis or impaired proliferation of β-cells as contributing factors to reduced insulin secretion [52–55]. We observed a reduced number of islets in cKO mice compared with the WT group (Fig. 3A), and this prompted us to investigate whether Talin-1 regulates β-cell apoptosis or proliferation. Firstly, we performed Terminal deoxynucleotidyl transferase dUTP nick end labeling (TUNEL) staining and found no significant difference in β-cell apoptosis between cKO mice and the WT mice (Fig. 3B, C). Then, we conducted IHC staining using specific insulin antibody following measurement of β-cell area/pancreatic ratio and β-cell mass, and found remarkable reductions in cKO mice compared to the respective WT groups (Fig. 3D–F). Next, we performed IHC staining on pancreatic sections using antibodies against the proliferation cell nuclear antigen (Pcna) and Cyclin D1, and observed reduced β-cell proliferation in cKO mice as both Pcna and Cyclin D1 were significantly decreased in cKO mice compared to that of the WT mice (Fig. 3G–J). Furthermore, mRNA levels of *Pcna* and *Ccnd1* were dramatically decreased in cKO islets compared to that of WT islets, while the expression of genes controlling β-cell differentiation and maturation, including *Mafa*, *Pdx1*, *Ngn3*, and *Nkx6.1* [29, 56–58], showed no significant differences (Fig. 3K). Consistently, IHC staining for MafA showed no significant difference between the two groups (Fig. 3L, M). Additionally, we knocked down Talin-1 expression in INS1 cells by siRNA and observed a significant reduction in Cyclin D1 protein levels while MafA expression was not altered (Fig. 3N, O). Besides, similar to changes of Talin-1 expression (Fig. 1), single cell analysis revelaed that genes involved in the progression from G1 to S phase were generally up-regulated in T1D and T2D, and b-cells showed significant higher level of S scores in diabetic diseases, which may be a compensatory mechanism of the body (Supplementary Fig. 2A, B).

Collectively, our findings suggest that Talin-1 deficiency reduces insulin expression mainly through inhibiting β-cell proliferation and mass which could contribute to the glucose intolerance of cKO mice.

### Talin-1 deficiency in β-cells decreases Stat3 expression

**Fig. 4:**
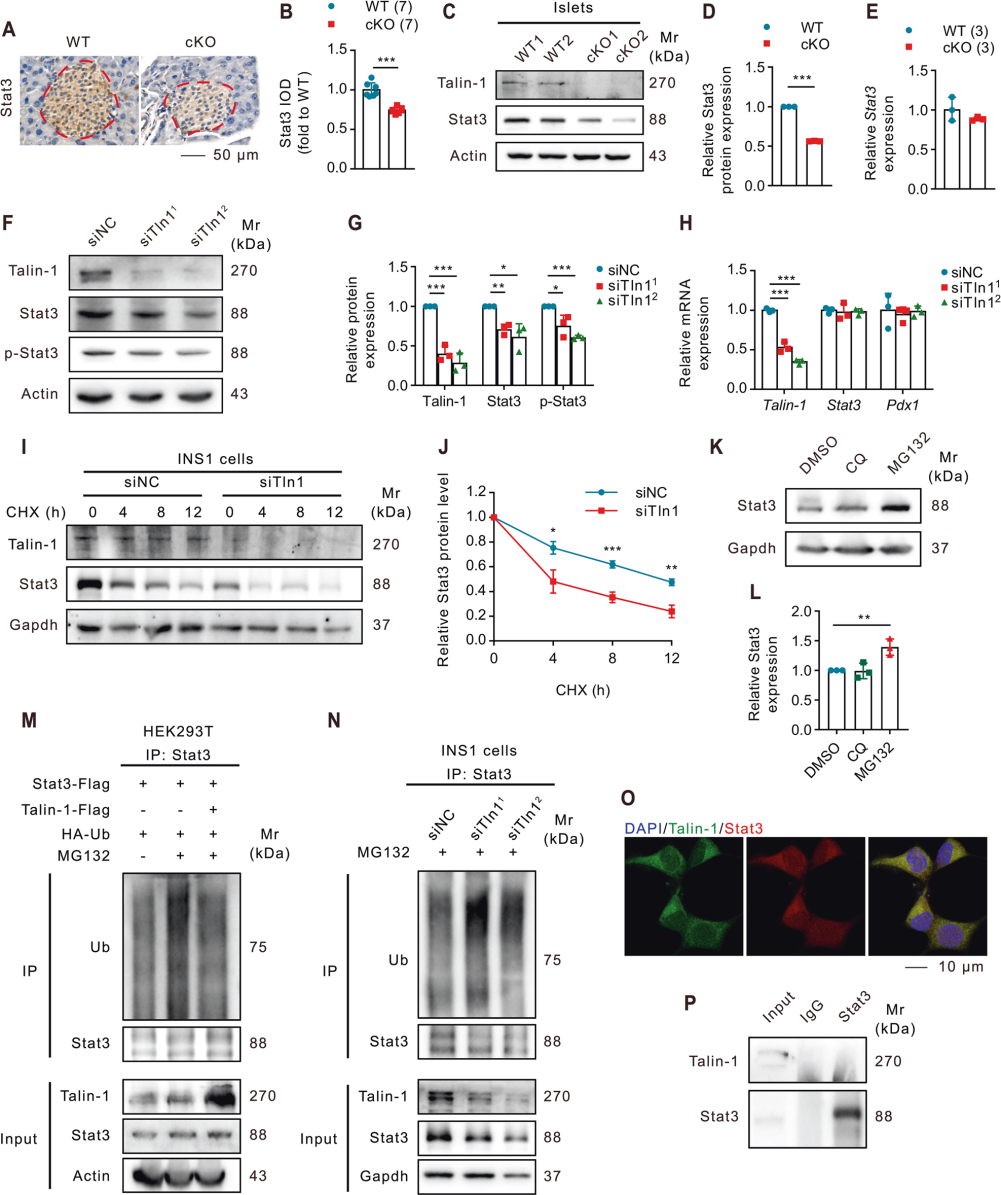
Talin-1 increases Stat3 protein stability by reducing its ubiquitination. **A**, **B** IHC staining for Stat3. Scale bar, 50 μm. Quantitative data (**B**). *N* = 7 mice per group. **C**, **D** Western blot analyses. Protein extracts isolated from the primary islets of 2-month-old male cKO and WT mice were subjected to western blot analyses for Stat3 expression. Actin was used as a loading control. Quantitative data (**D**). **E** QPCR analyses. Total RNAs isolated from the primary islets of 2-month-old male cKO and WT mice were subjected to qPCR for *Stat3* expression. *Stat3* mRNA was normalized to *Gapdh* mRNA. **F**, **G** Western blot analyses. INS1 cells were co-transfected with the plasmids of siTln1 for 48 h and next the protein extracts were subjected to western blot analyses with indicated antibodies. Quantitative data (**G**). **H** QPCR analyses. Total RNAs isolated from INS cells, after co-transfected with the plasmids of siTln1, were subjected to qPCR for *Talin-1*, *Stat3*, and *Pdx1* expression. All mRNA was normalized to *Gapdh* mRNA. **I**, **J** Cycloheximide (CHX) experiments. INS1 cells with and without siTln1 knockdown were treated with 100 μg/mL of CHX for the indicated times, followed by western blot analyses for expression of Stat3. Quantitative data from three independent experiments (**J**)**. K**, **L** Western blot analyses. Effect of MG132 and Chloroquine (CQ) on Stat3 protein expression in INS1 cells. Quantitative data (**L**)**. M** Talin-1 overexpression decreases Stat3 ubiquitination. HEK293T cells were transiently transfected with Stat3 plasmid with or without Talin-1 plasmid. At 48 h after the transfection, cells were treated with or without MG132 (10 μM) for 6 h, then immunoprecipitation (IP) and immunoblotting (IB) with the indicated antibodies were performed. **N** Talin-1 knockdown increases endogenous Stat3 ubiquitination. INS1 cells were transiently transfected with control siRNAs or siTln1. At 48 h after transfection, cells were treated with MG132 (10 μM) for 6 h, then IP and IB with the indicated antibodies were performed. **O** IF staining. INS1 cells were subjected to double immunostaining with an anti-Stat3 antibody (red) and an anti-Talin-1 antibody (green). Scale bar, 10 μm. **P** IP assays. INS1 cells were transiently transfected with Talin-1 and the cell lysates were collected for IP and IB with the indicated antibodies. Results are expressed as mean ± standard deviation (s.d.) and unpaired two-tailed Student’s *t* test (two groups) or one-way ANOVA (multiple groups) was performed to analyze the difference between the two groups. *p < 0.05, **p < 0.01, ***p < 0.001, versus control.

Signal transducer and activator of transcription 3 (Stat3) has been recognized as a indispensable modulator of pancreatic β-cell development and function, and has been implicated in regulating β-cell cycle and protecting β-cells from DNA damage in chronic pancreatitis [59–62]. Our single cell analysis also suggested that Stat3 might play potential roles in regulating cell cycle of β-cells (Supplementary Fig. 2). Therefore, we performed IHC staining to determine the expression of Stat3 in pancreatic sections and found that Stat3 signal was significantly reduced in cKO islets compared to that of WT mice (Fig. 4A, B). Consistently, the protein level of Stat3 was also markedly decreased in cKO islets compared to that of WT control islets revealed by WB analyses (Fig. 4C, D). However, there was no significant differences in *Stat3* mRNA expression between islets from cKO and WT mice (Fig. 4E). To further validate these findings, we knocked down Talin-1 expression in INS1 cells and observed that Talin-1 knockdown significantly reduced the protein levels of both total and p-Stat3 without affecting *Stat3* mRNA expression (Fig. 4F–H), which is consistent with the in vivo results. The above results suggest that Talin-1 deficiency reduces Stat3 protein level but not mRNA expression.

### Talin-1 regulates Stat3 protein stability by modulating its ubiquitination

Next, we investigated possible mechanisms by which Talin-1 regulates Stat3 protein level. We conducted cycloheximide (CHX) experiments to examine if Talin-1 knockdown could affect Stat3 protein stability in INS1 cells and found that knockdown of Talin-1 significantly accelerated Stat3 protein degradation (Fig. 4I, J). To determine the degradation pathway involved in Stat3 protein degradation, we treated INS1 cells with MG132 and Chloroquine (CQ), which inhibits the proteasome and lysosome pathways, respectively. WB analyses demonstrated that MG132 treatment significantly increased Stat3 protein accumulation, while CQ treatment had no effect on Stat3 protein level (Fig. 4K, L), indicating that Stat3 degradation is mediated primarily through the proteasome pathway in INS1 cells. Furthermore, overexpression of Talin-1 in HEK293T cells led to a significant reduction of Stat3 polyubiquitination (Fig. 4M). In contrast, Talin-1 knockdown in INS1 cells enhanced the polyubiquitination of endogenous Stat3 protein (Fig. 4N). These results suggest that Talin-1 maintains Stat3 protein stability through inhibiting Stat3 ubiquitination and proteasomal degradation.

### Talin-1 inhibits Smurf1-mediated Stat3 ubiquitination

**Fig. 5:**
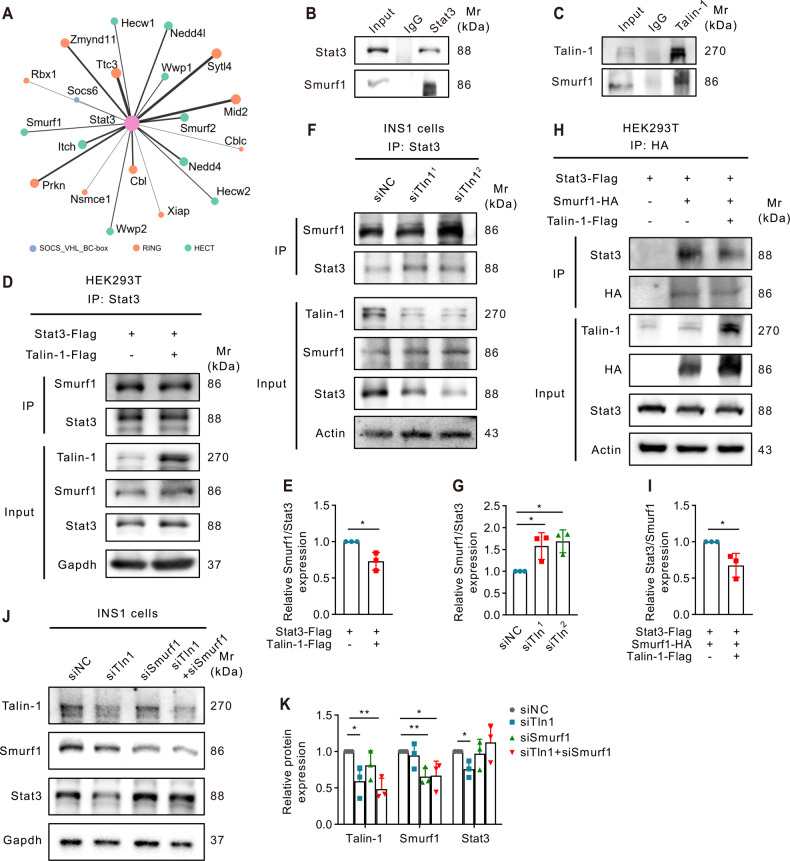
Talin-1 enhances Stat3 protein stability through inhibiting Smurf1-dependent Stat3 ubiquitination and proteasomal degradation. **A** Top 20 predicated mouse E3 ubiquitin ligases which could potentially interact with Talin-1. **B**, **C** IP assays. INS1 cells were transiently transfected with Talin-1 and the cell lysates were collected for IP and IB with the antibodies as indicated. **D**, **E** Western blot analyses. Talin-1 overexpression reduced the interaction between Smurf1 and Stat3. HEK293T cells transfected with the indicated plasmids were subjected to IP and IB assays with the antibodies as indicated. Quantitative data from three independent experiments (**E**)**. F**, **G** Western blot analyses. Talin-1 knockdown increased the interaction between Smurf1 and Stat3. Cell lysates from INS1 cells transfected with siTln1 or negative control siRNA (siNC) were subjected to IP and IB assays with the indicated antibodies. Quantitative data from three independent experiments (**G**)**. H**, **I** Western blot analyses. Talin-1 overexpression reduced the binding affinity between Smurf1 and Stat3. HEK293T cells transfected with the indicated plasmids were subjected to IP and IB assays with the indicated antibodies. **J**, **K** Western blot analyses. INS1 cells were transiently transfected with siTln1 or siSmurf1 or siTln1+siSmurf1. Cell lysates were subjected to western blot analyses with the indicated antibodies. Quantitative data from three independent experiments (**K**).

Based on the aforementioned findings, we performed IF staining and observed the co-localization of Talin-1 and Stat3 in INS1 cells (Fig. 4O). However, no direct protein interaction was detected between Talin-1 and Stat3 by co-immunoprecipitation (co-IP) assays (Fig. 4P). This prompted us to speculate that an E3 ligase may be involved in regulating the ubiquitination and degradation of Stat3 protein by directly interacting with Talin-1. To identify potential E3 ubiquitin ligases involved in Stat3 degradation, we conducted bioinformatics analysis using UbiBrowser, which predicted the top 20 E3 ubiquitin ligases with higher confidence levels (Fig. 5A). Additionally, we predicted protein-protein interactions of Talin-1 using HitPredict and identified smad ubiquitination regulatory factor 1 (Smurf1) as the only E3 ligase that was predicted to interact with both Stat3 and Talin-1. Subsequently, we performed co-IP assays to validate the predicted interactions between Smurf1, Stat3, and Talin-1 in INS1 cells, and as expected, we observed interactions between Smurf1 and Stat3 (Fig. 5B), Smurf1 and Talin-1 (Fig. 5C). We next investigated the potential involvement of Smurf1 in the degradation of Stat3 protein regulated by Talin-1. Results showed that when Talin-1 was overexpressed, the binding affinity between Smurf1 and Stat3 was reduced (Fig. 5D, E). In contrast, when Talin-1 was knocked down, the interaction between Smurf1 and Stat3 was enhanced (Fig. 5F, G). Moreover, Smurf1 overexpression significantly enhanced the binding affinity between Smurf1 and Stat3, which was attenuated by Talin-1 overexpression (Fig. 5H, I). Finally, Smurf1 knockdown was able to reverse the decreased level of Stat3 caused by Talin-1 knockdown in INS1 cells (Fig. 5J, K). These findings suggest that Talin-1 plays a role in modulating Smurf1-dependent Stat3 ubiquitination and degradation. We further performed IHC staining of pancreatic sections and found that no significant differences in the expression of Smurf1 between WT and cKO mice (Supplementary Fig. 3).

### Haploinsufficiency of *Tln1* and *Stat3* genes in β-cells causes glucose intolerance in mice

**Fig. 6:**
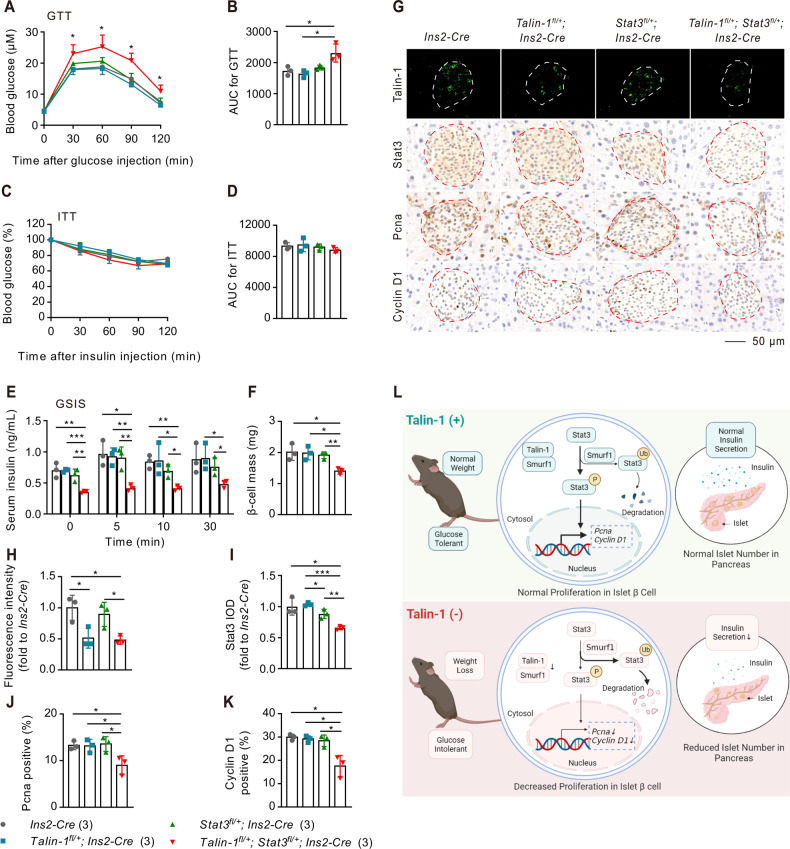
Haploinsufficiency of *Talin-1* and *Stat3* genes leads to diabetes-like phenotypes in mice. **A** GTT. 8-week-old male mice were subjected to GTT. **B** AUC calculated based on (**A**). **C** ITT. 9-week-old male mice were subjected to ITT. **D** AUC was calculated based on (**C**). **E** GSIS. 10-week-old male mice were subjected to GSIS. **F** β-cell mass measurements. **G–K** IF and IHC staining for Talin-1, Stat3, Pcna, and Cyclin D1. Scale bar, 50 μm. Quantitative data (**H–K**). **L** Graphic abstract depicting a proposed model for Talin-1/Smurf1/Stat3 axis in modulating β-cell proliferation. *N* = 3 mice per group. Results are expressed as mean ± standard deviation (s.d.) and unpaired two-tailed Student’s *t* test (two groups) or one-way ANOVA (multiple groups) was performed to analyze the difference between the two groups. *p < 0.05, **p < 0.01, ***p < 0.001, versus control.

We further investigated the role of Stat3 in mediating the effect of Talin-1 defficiency on glucose intolerance by deleting one allele of *Talin-1* and/or *Stat3* genes specifically in β-cells with the same *Ins2-Cre* mice, and we obtained *Talin-1* or *Stat3* singly heterozygous mice (i.e., *Talin-1*^*fl/+*^*; Ins2-Cre* or *Stat3*^*fl/+*^*; Ins2-Cre*) and double heterozygous mice (i.e., *Talin-1*^*fl/+*^*; Stat3*^*fl/+*^*; Ins2-Cre*). Body weight monitoring in mice showed no significant difference in the growth among four genotypes (Supplementary Fig. 4A). GTT assays of two-month-old male mice of these three genotypes, along with *Ins2-Cre* mice as controls, revealed that either *Talin-1*^*fl/+*^*; Ins2-Cre* or *Stat3*^*fl/+*^*; Ins2-Cre* mice did not show obvious differences in blood glucose clearance compared to the control *Ins2-Cre* mice, however, the ability of glucose clearance of *Talin-1*^*fl/+*^*; Stat3*^*fl/+*^*; Ins2-Cre* mice was significantly decreased compared to that of the *Ins2-Cre* mice (Fig. 6A, B). Additionally, ITT assays showed no significant difference in peripherial insulin sensitivity among the four groups (Fig. 6C, D). GSIS assays demonstrated impaired insulin secretion in *Talin-1* and *Stat3* double heterozygous mice compared to that of singly heterozygous or control mice (Fig. 6E). Moreover, β-cell mass was also markedly decreased in *Talin-1*^*fl/+*^*; Stat3*^*fl/+*^*; Ins2-Cre* mice compared with that of the other three groups (Fig. 6F). IHC staining showed that *Talin-1*^*fl/+*^*; Stat3*^*fl/+*^*; Ins2-Cre* mice had less Pcna or Cyclin D1 positive β-cells compared to the other three groups (Fig. 6G–K). TUNEL assays indicated no significant difference in apoptosis of β-cells among the four groups (Supplementary Fig. 4B). There was no significant difference in the expression of Smurf1 among four groups demonstrated by IHC staining (Supplementary Fig. 4C, D).

Collectively, the above findings of our study provide the first evidence that Talin-1 and Stat3 function in the same genetic pathway to regulate the proliferation of islet β-cells. Specifically, findings from our study suggests that Talin-1 prohibits the ubiquitination of Stat3 by Smurf1 through interaction with Smurf1, thereby increases β-cell proliferation and mass, insulin expression, and thus maintains glucose homeostasis (Fig. 6L).

### Talin-1 deficiency in β-cell exerbates HFD-induced glucose intolerance without affecting peripherial insulin sensitivity

**Fig. 7:**
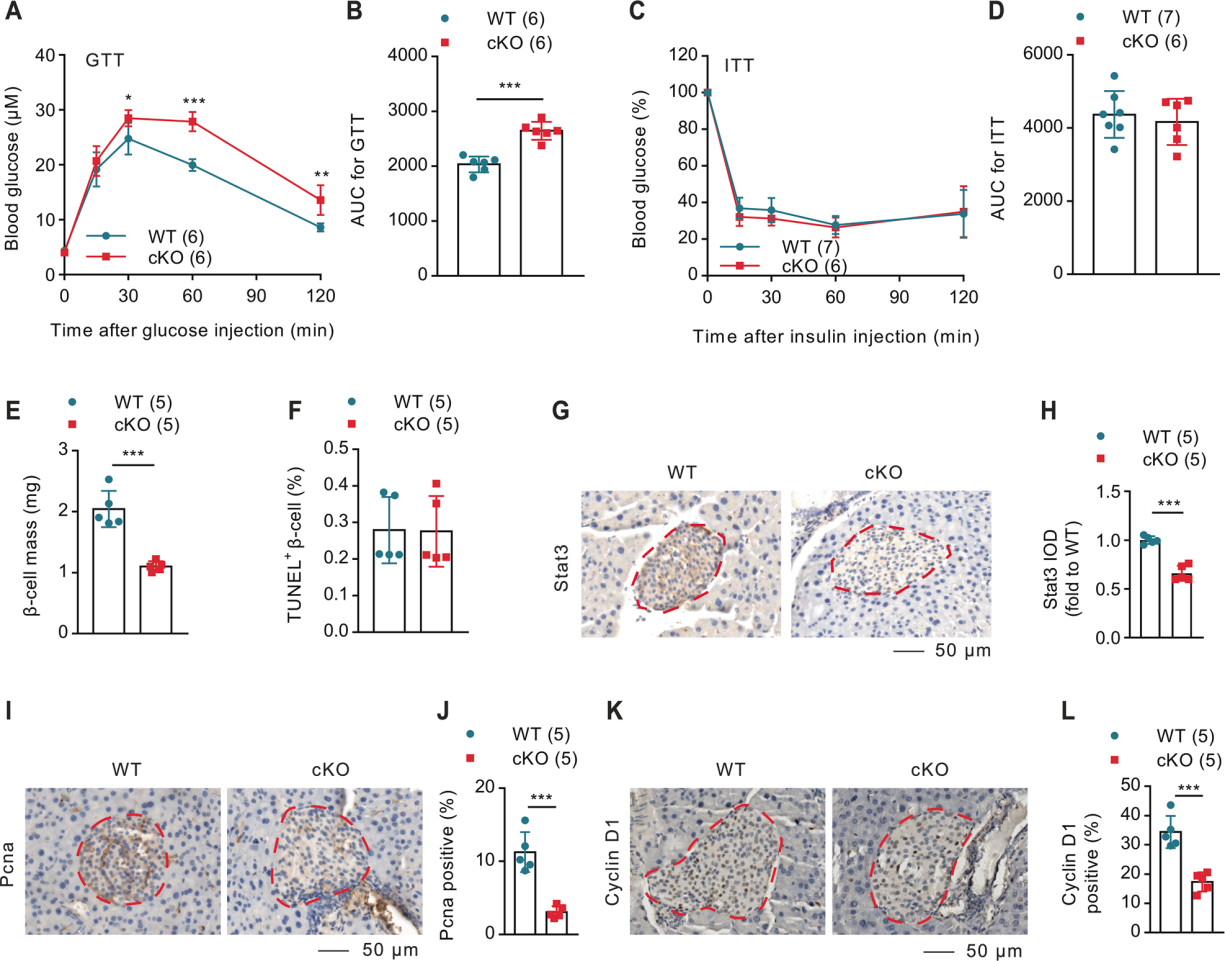
High-fat diet exacerbates blood glucose intolerance without affecting insulin sensitivity in cKO mice. **A** GTT. 12-week-old male mice (Treated with HFD for 6 weeks) were subjected to GTT. **B** AUC calculated based on (**A**). *N* = 6 mice per group. **C** ITT. 13- week-old male mice (Treated with HFD for 7 weeks) were subjected to ITT. **D** AUC calculated based on (**C**). *N* = 7 mice for WT and *N* = 6 mice for cKO. **E** β-cell mass for HFD-fed WT and cKO mice. *N* = 5 mice per group. **F** TUNEL staining. Analysis of HFD-fed WT and cKO mice for the percentage of TUNEL positive β-cells. *N* = 5 mice per group. **G–L** IHC staining for Stat3, Pcna and Cyclin D1 on pancreatic sections from HFD-fed WT and cKO mice. Scale bar, 50 μm. *N* = 5 mice per group. Results are expressed as mean ± standard deviation (s.d.) and unpaired two-tailed Student’s *t* test (two groups) or one-way ANOVA (multiple groups) was performed to analyze the difference between the two groups. *p < 0.05, **p < 0.01, ***p < 0.001, versus control.

Next, we examined whether Talin-1 plays a critical role in preserving β-cell functions under stressful conditions. We challenged 6-week-old cKO and WT mice with a high-fat diet (HFD), a well-established model to induce β-cell stress [50, 51], and determined whether β-cell function in cKO mice would be more severely compromised compared to WT mice. After 7 weeks HFD treatment, we performed GTT and observed a significant increase in blood glucose levels in cKO mice compared to sex- and age-matched WT mice, persisting from 15 to 120 min (Fig. 7A, B), and this increase was more significant than under normal chow dietary (NCD) conditions (Fig. 2B, C). Similarly, after 7 weeks of HFD treatment, there were no significant differences in peripherial insulin sensitivity between cKO mice and the WT control mice (Fig. 7C, D). Notably, these findings strongly suggest that Talin-1 deficiency in β-cells further impairs glucose homeostasis under stressful conditions. We also performed IHC staining using specific antibody against insulin to measure β-cell mass and found remarkable reductions in cKO mice compared to the respective WT groups (Fig. 7E). TUNEL staining showed no significant difference in β-cell apoptosis between cKO mice and the WT control mice (Fig. 7F). Consistently, IHC staining showed that Stat3 signal, Pcna and Cyclin D1 were all significantly reduced in cKO islets compared to that of WT mice (Fig. 7G–L), while Smurf1 expression was not obviously changed between the two groups (Supplementary Fig. 5A, B).

### Talin-1 deficiency in adult mice causes glucose intolerance

**Fig. 8:**
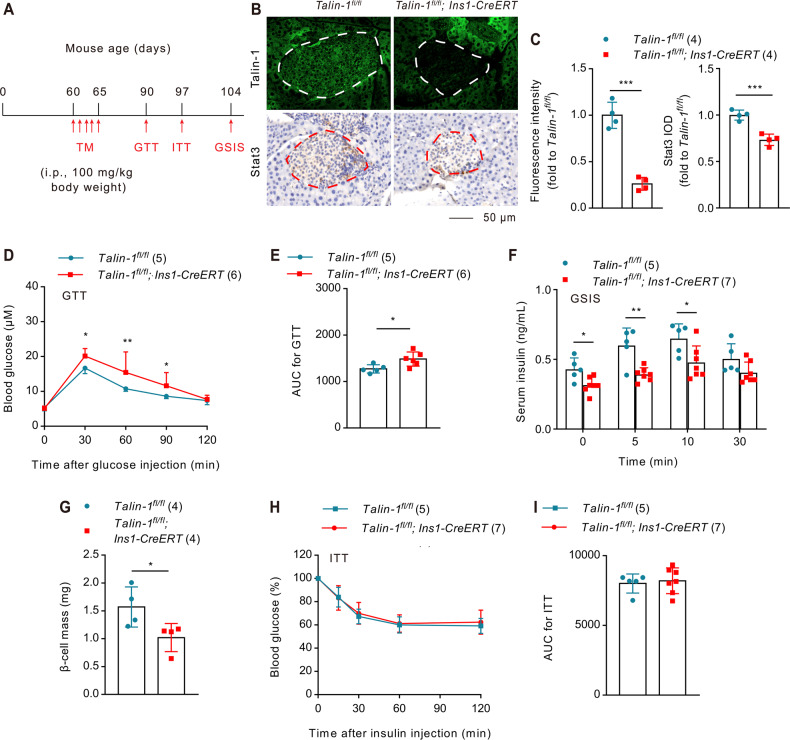
Inducible deletion of β-cell Talin-1 in adult mice causes diabetes-like phenotypes. **A** Tamoxifen (TM) injection. **B**, **C** IF and IHC staining. Two-month-old male *Talin-1*^*fl/fl*^*; Ins1-CreERT* and control (*Talin-1*^*fl/fl*^) mice were treated with TM as described in (**A**). After 30 days, Talin-1 and Stat3 were detected by IF and IHC respectively using pancreatic sections. Scale bar, 50 μm. Quantitative data (**C**). *N* = 4 mice per group. **D** GTT. GTT assays were performed 30 days after the first TM injection shown in (**A**). **E** AUC is calculated based on (**D**). *N* = 5 mice for *Talin-1*^*fl/fl*^ mice and *N* = 6 mice for *Talin-1*^*fl/fl*^*;Ins1-CreERT* mice. **F** GSIS. Mice were treated with TM as in (**A**). GSIS assays were performed 44 days after the first TM injection. *N* = 5 mice for *Talin-1*^*fl/fl*^ mice and *N* = 7 mice for *Talin-1*^*fl/fl*^*;Ins1-CreERT* mice. **G** β-cell mass measurements of pancreatic sections from *Talin-1*^*fl/fl*^*; Ins1-CreERT* and control (*Talin-1*^*fl/fl*^) mice. *N* = 4 mice per group. **H** ITT. Mice were treated with TM as in (**A**). ITT assays were performed 37 days after the first TM injection. **I** AUC is calculated based on (**H**). *N* = 5 mice for *Talin-1*^*fl/fl*^ mice and *N* = 7 mice for *Talin-1*^*fl/fl*^*;Ins1-CreERT* mice. Results are expressed as mean ± standard deviation (s.d.) and unpaired two-tailed Student’s *t* test (two groups) or one-way ANOVA (multiple groups) was performed to analyze the difference between the two groups. *p < 0.05, **p < 0.01, ***p < 0.001, versus control.

We further examined the impact of Talin-1 deficiency on glucose homeostasis in adult mice by using mice bearing conditional alleles of *Talin-1* (*Talin-1*^*fl/fl*^) and an *Ins1-CreERT* transgene, which allows for tamoxifen (TM)-inducible Cre recombinase expression specifically in β-cells. These mice (2-month-old) were treated with injections of TM (100 mg/kg body weight) for five days to delete Talin-1 expression in β-cells (hereafter referred to as *Talin-1*^*fl/fl*^*; Ins1-CreERT*) (Fig. 8A). Similar to results from *Talin-1*^*fl/fl*^*; Ins2-Cre* mice, IF and IHC staining confirmed a marked reduction in Talin-1 and Stat3 protein expression in the islets of *Talin-1*^*fl/fl*^*; Ins1-CreERT* mice relative to that of *Talin-1*^*fl/fl*^ control littermates (Fig. 8B, C). At day 35 after TM injections, *Talin-1*^*fl/fl*^*; Ins1-CreERT* mice exhibited significant impairment in glucose clearance (Fig. 8D, E) and insulin secretion indicated by GTT and GSIS assays, respectively (Fig. 8F). Furthermore, there was a marked reduction in β-cell mass in *Talin-1*^*fl/fl*^*; Ins1-CreERT* mice (Fig. 8G). And also, no significant difference in peripherial insulin sensitivity was observed between the two groups (Fig. 8H, I). Consistently, we performed TUNEL staining and found no significant differences in β-cell apoptosis between *Talin-1*^*fl/fl*^*; Ins1-CreERT* mice and *Talin-1*^*fl/fl*^ mice (Supplementary Fig. 5C). Moreover, IHC staining also indicated no significant difference in the expression of Smurf1 between *Talin-1*^*fl/fl*^*; Ins1-CreERT* mice and *Talin-1*^*fl/fl*^ mice (Supplementary Fig. 5D, E).

Collectively, these results further indicate the importance of Talin-1 in controlling β-cell proliferation and glucose homeostasis and may serve as a potential therapeutic target for diabetes milletus.

## Discussion

Our study elucidates the significant role of Talin-1/Smurf1/Stat3 axis in the regulation of glucose homeostasis through modulating β-cell proliferation in islets. Talin-1 defficiency in β-cells leads to hyperglycemia in mice upon glucose challenge, which is primarily attributed to impaired glucose-stimulated insulin secretion (GSIS) and reduced β-cell mass, rather than peripheral insulin resistance. Reduce β-cell mass and decreased insulin secretion are key pathologic events for diabetes. Findings from this study are of great significance which highlight the potential therapeutic strategy by targeting the local Talin-1/Smurf1/Stat3 axis to mitigate β-cell mass loss and enhance insulin secretion in patients with diabetes or hyperglycemia, offering potential avenues for intervention in these conditions.

In our study, we for the first time demonstrate the potential protective role of Talin-1 in the regulation of blood glucose homeostasis, which is supported by multiple lines of evidence, including: (1) upregulation of Talin-1 expression in islet β-cells of diabetes patients, as well as in islets of aged and HFD-fed mice; (2) Talin-1 deficiency impairs β-cell proliferation, resulting in reduced insulin secretion and glucose intolerance in mice; (3) Talin-1 deficiency exerbates glucose intolerance in HFD-fed cKO mice; (4) inducible deletion of Talin-1 in islet β-cells in adult mice causes severe glucose intolerance compared to WT control mice. These findings suggest that Talin-1 may undergo compensatory upregulation under diseased conditions.

Talin-1 deficiency impairs β-cell proliferation at least in part by decreasing Stat3 protein, resulting in reduced β-cell mass and insulin secretion, and glucose intolerance in mice. Stat3 is a transcription factor known to regulate gene expression involved in cell growth and proliferation, including Pcna, Cyclin D1, etc. [14, 15, 62–65]. Our in vivo study shows that Stat3 is dramatically decreased in β-cells in mice with β-cell specific deletion of Talin-1 both during development and homeostasis. Our in vitro study also shows that Talin-1 knockdown by siRNA reduces Stat3 protein level. Furthermore, we for the first time demonstrate from in vivo that Talin-1 and Stat3 functions in the same genetic pathway in β-cells to regulate glucose homeostasis in mice, and mice of double heterozygous for *Talin-1* and *Stat3* in islet β-cells display severe glucose intolerance, impaired GSIS and reduced β-cell mass compared to either singly heterozygous or control mice.

In present study, we demonstrate that Talin-1 modulates Stat3 protein level in β-cells at least partially through Smurf1, a member of the E3 ubiquitin ligase family, which plays crucial roles in modulating cell differentiation, proliferation, apoptosis, etc. by modulating protein ubiquitination and degradation [66–70]. No physical interaction between Talin-1 and Stat3 is detected in this study, indicating the indirect regulation of Stat3 by Talin-1. However, we for the first time demonstrate that Smurf1 physically interacts with Stat3 protein and regulates Stat3 ubiquitination and subsequent degradation, thereby modulating the expression of key factors downstream of Stat3 such as Pcna and Cyclin D1, which are involved in controlling cell proliferation. Interestingly, Bian et al. reported that Smurf1-mediated ubiquitylation of SH2 domain-containing phosphatase 1 (SHP-1) promoted endometrial stromal cell proliferation and invasion in endometrial stromal cells through activation of Stat3 signaling [71]. Thus, our study and others demonstrate the key role of Smurf1 in modulating Stat3 protein level through direct or indirect methods. Furthermore, we find that Talin-1 also physically interacts with Smurf1, which prohibits the interaction between Smurf1 and Stat3, thus promoting Stat3 accumulation. Our in vitro experiments demonstrate that Talin-1 knockdown increases the binding affinity between Smurf1 and Stat3, and results in increased Stat3 ubiquitination and protesomal degradation. In contrast, Talin-1 overexpression reduces the interaction between Smurf1 and Stat3, and results in decreased Stat3 ubiquitination and proteasomal degradation. Thus, Talin-1 can be regarded as a gripper, grasping Smurf1, preventing Smurf1 from binding to Stat3 and making it ubiquitinated and degraded.

The reduced β-cell mass of mice with Talin-1 loss in β-cells is due to reduced β-cell proliferation, but not β-cell differentiation. The expression of genes that regulate β-cell differentiation, including *Pdx1, Ngn3, Nkx6.1, MafA*, etc., are not affected by Talin-1 deletion in β-cells. Both mRNA and protein level of MafA, a master regulator that control insulin gene transcription, are not significantly affected by Talin-1 defficiency. These findings together with the normal in vitro GSIS suggest that insulin expression per β-cell and the secretion process are probably not significantly affected, and the decreased glucose clearance and in vivo GSIS is primarily due to the reduced β-cell mass caused by reduced β-cell proliferation in mice with Talin-1 loss in β-cells.

In conclusion, our study for the first time demonstrate the novel role of Talin-1 in regulating the proliferation of pancreatic islet β-cells and its impact on glucose homeostasis in mice. Specifically, Talin-1 functions to maintain β-cell proliferation by mitigating Stat3 ubiquitination and proteasomal degradation mediated by Smurf1. Modulating the Talin-1/Smurf1/Stat3 axis in pancreatic islet β-cells could serve as a promising therapeutic approach for patients with diabetes.

## Materials and methods

### Bioinformatics

The Seurat object of human islets scRNA-seq data, which contained 222, 077 cells from 31 individuals of healthy control, 10 patients of autoantibody (AAB), 9 patients of T1D, and 17 patients of T2D, was downloaded from the Data portal of The Human Pancreas Analysis Program (PANC-DB). To remove potential cells of fractions, dead cells, or other contaminants, we performed quality control and excluded clusters with a median feature number lower than 600 or a median UMI count lower than 2000. After unsupervised clustering and cell type annotations, the remaining quality-passed cells were identified as alpha cells (*GCG*, *IRX2*, *PLCE1*, *LOXL4*), beta cells (*INS*, *IAPP*, *DLK1*, *NPTX2*), delta cells (*SST*, *PRG4*, *LEPR*, *RBP4*), Pancreatic polypeptide (PP) cells (*SERTM1*, *PPY*, *ENTPD2*, *ETV1*), acinar cells (*PRSS1*, *PNLIP*, *CTRB2*, *CELA3A*), duct cells (*SPP1*, *CFTR*, *KRT10*, *AQP1*), and stromal cells (*COL1A1*, *MGP*, *DCN*). To compare the proportion of each cell type in different diseases with healthy control, the *stat_compare_means* function implemented in the R package was employed.

The cell cycle score was calculated based on expression levels of genes associated with S-phase and G2M-phase. And genes involved in the progression from G1 to S phase was downloaded from the UniProt database.

The Wilcoxon rank sum test was adopted in all statistics, and the *p*-value was adjusted with the Bonferroni method.

We used Ubibrowser to predict the E3 ligases for Stat3. HitPredict was used to predict the physical protein-peotein interaction.

### Mice

The generation of *Talin-1*^*fl/fl*^ mice was previously reported [72, 73]. To specifically delete Talin-1 expression in β-cells, we bred the *Talin-1*^*fl/fl*^ mice with *Ins2-Cre* transgenic mice harboring the rat insulin II gene promoter (*RIP*) that drives Cre recombinase expression in β-cells (Jackson laboratory, Bar Harbor, ME, USA), and generated β-cell selective Talin-1 knockout mice (*Talin-1*^*fl/fl*^*; Ins2-Cre* or cKO mice). To delete Talin-1 in β-cells of adult mice, we bred *Talin-1*^*fl/fl*^ mice with *Ins1-Cre/ERT* transgenic mice and generated inducible conditional Talin-1 knockout mice (*Talin-1*^*fl/fl*^*; Ins1-CreERT*). Tamoxifen (Sigma, T5648) was intraperitoneally injected to 2-month-old mice daily for 5 days (100 mg/kg body weight). *Stat3*^*fl/fl*^ mice were described previously [61], and were kindly shared from X.-Y. Fu of Indiana University School of Medicine. To generate mice with haploinsufficiency of *Talin-1* and *Stat3* genes, we bred *Stat3*^*fl/fl*^ mice with *Talin-1*^*fl/fl*^*; Ins2-Cre* mice or C57BL/6J mice to obtain *Stat3*^*fl/+*^*; Talin-1*^*fll+*^*; Ins2-Cre* mice, *Stat3*^*fl/+*^*; Ins2-Cre* mice, *Talin-1*^*fll+*^*; Ins2-Cre* mice, and other genotypes of mice for this study. All animal models used in this study were maintained on a C57BL/6J background and littermatesb with weight-matched were used in a randomized way. The sample size of each experiment was chosen based on previous experimental observations and indicated in the figure legend. Mice were housed at 20–24 °C on a 12 h light/dark cycle. In this study, for data consistency and to minimize the use of mice, only male mice were used for all experiments. All animal protocols used in this study were approved by the Institutional Animal Care and Use Committees of the Southern University of Science and Technology.

### Human pancreatic samples

Human pancreatic sections containing pancreatic cancer adjacent tissues were provided by Shanghai Outdo Biotech (HPanA180Su10), and were used for IHC staining to examine Talin-1 expression. The informed consent was obtained from all subjects. The study protocol was approved by the ethics committee of Shanghai Outdo Biotech (YB M-05-02).

### Cells

Rat INS1 832/13 cells from ATCC (provided by Prof. Feng Rao, Southern University of Science and Technology, Shenzhen, China) were cultured in a humidified atmosphere of 5% CO_2_/95% air at 37 °C in RPMI-1640 medium (GIBCO^TM^, C11875500BT) containing 10% fetal bovine serum (FBS; GIBCO^TM^, 10091148), 100 U/mL penicillin and 100 μg/mL streptomycin.

### Glucose tolerance test (GTT) and insulin tolerance test (ITT) and Glucose-stimulated insulin secretion assay (GSIS)

After fasted for 16 h overnight, mice were performed i.p. injection of glucose (2 g/kg of body weight) for GTTs. Blood glucose levels were measured at 0, 15, 30, 60, and 120 min after glucose injection using a blood glucose meter (YASEE, GLM-76). ITTs were performed on mice fasted for 4 h by i.p. injection of recombinant human insulin (ProSpec, cyt-270) at a dose of 1 U/kg of body weight. Blood glucose levels were measured at 0, 15, 30, 60, and 120 min after insulin injection. For GSIS, mice were fasted for 16 h and then received glucose (2 g/kg of body weight) by i.p. injection. Blood samples were collected from tail veins at 0, 5, 15, and 30 min after glucose injection. ELISA kits (EZassay, MS200) were used to determine blood insulin levels. GTT, ITT, and GSIS were performed and analyzed in a double-blinded way.

### Isolated islets insulin secretion assay

Islets were isolated following the protocol of Li et al. [74] Briefly, mice were sacrificed by cervical dislocation. 30 G needles were used to inject 3 ml cold collagenase V (Sigma, C9263) solution [1 mg/mL in Hanks buffered salt solution (HBSS)] into the common bile duct until the pancreas was visibly distended. Pancreas were removed and digested at 37 °C for 15 min, and the digestion was terminated by adding 10 ml ice-cold Hanks buffer containing 0.2% bovine serum albumin (BSA; Sigma, A8806). Islets were hand-picked and cultured with RPMI-1640 medium containing 10% FBS, 100 U/mL penicillin, and 100 μg/mL streptomycin.

Isolated islets were preincubated in 96-well plates for 2.5 h in RMPI-1640 medium containing 10% FBS and 2.5 mM glucose at 37 °C and 5% CO_2_. Then islets were incubated in a fresh medium containing 16.7 mM glucose solutions for 30 min. Then islets and supernatant were collected after treatment. The total protein in islets was extracted by sonication in 300 μl acid/ethanol (0.18 M HCl in 95% ethanol). The levels of insulin in the collected supernatant and extracts were detected using an insulin ELISA kit (EZassay, MS200) following the manufacturer’s instruction.

### High-fat diet (HFD) treatment

At the age of 5 weeks, mice were fed with HFD (Research, D12492), which consists of a Rodent Diet with 60% kcal fat. GTT, ITT, and GSIS were performed after HFD treatment for 6, 7, and 8 weeks, respectively. HFD treatment lasted for 9 weeks, with body weights monitored weekly.

### β-cell mass

β-cell mass were calculated as previous reported [29, 61]. Pancreases were processed for paraffin sections, and at least 6 five-micrometer sections throughout the entire pancreas were obtained from each mouse. All pancreatic sections were subjected to immunohistochemical (IHC) staining for insulin. A multifunctional digital pathology scanner (Aperio VERSA 8) was used to scan the total area of each slide. β-cell ratio was obtained by dividing total insulin-positive area by total pancreatic area. β-cell mass was calculated by multiplying the β-cell ratio with the pancreas weight.

### Quantitative real-time RT-PCR (qPCR)

Total RNA was extracted from cells or tissues using Trizol regent (TransGen Biotech, ET111-01), and then reverse transcribed into cDNA using the Titanium One-Step RT-PCR Kit (Takara, 639504) according to manufacturer’s protocols. Quantitative real-time RT-PCR (qPCR) analysis was applied to measure the relative mRNA levels using SYBR Green dye (Bio-Rad, 1725125) in the Analytik Jena AG qTOWER3 Real-time PCR system. Results were normalized to *Gapdh* expression and processed using the ΔΔCT method. All DNA sequences of primers used for qPCR were listed in Supplementary Tables 1 and 2.

### Western blot analyses

Western blot analyses were performed as we previously described [75]. Briefly, cells or tissue proteins were harvested in RIPA lysis buffer with 0.1% protease inhibitor cocktail (YEASEN, 20124ES10). Protein extracts were fractionated on a 10% SDS-PAGE gel and transferred onto PVDF membranes (Thermo Scientific™, 88518). Then, the membranes were blocked in 5% nonfat milk in Tris-buffered saline/Tween 20 buffer for 2 h at room temperature, probed with primary antibodies overnight at 4 °C, followed by incubation with secondary antibodies conjugated with horseradish peroxidase for 2 h at room temperature, and visualized using a Western Blotting Detection Kit (Mei5bio, MF-078-01) by a chemiluminescence imaging system (LAS4000, ImageQuant). All antibodies used in this study were summarized in Supplementary Table 3.

### Immunohistochemistry (IHC) and Immunofluorescence (IF) staining

IHC and IF were performed as previously reported [75]. For IHC, five-micrometer-thick sections from formalin-fixed and paraffin-embedded tissues were incubated with primary antibodies at 4 °C overnight, followed by incubation with secondary antibodies. Chromogen development was determined by DAB Kit (Vectorlabs, SK-4100). A Multifunctional digital pathology scanner (Aperio VERSA 8) was used to scan the total area of each slide. For IF, 5 μm sections were permeabilized with 0.2% Triton X-100 containing DAPI for 5 mins and then blocked with 2% BSA for 1 h. Samples were next stained with primary antibodies (Supplementary Table 3) overnight at 4 °C. After washed with PBS, samples were incubated with corresponding secondary antibodies for 1 h at room temperature. Lastly, slides were imaged using a confocal microscope (Zeiss, LSM 980).

### Immunoprecipitation (IP), ubiquitination assay, and cycloheximide (CHX) assay

IP assays were performed as previously described [76]. Briefly, cultured HEK293T or INS1 cells were transiently co-transfected with the plasmids of interest using transfection regent (Thermo) following the manufacturer’s instructions. After 48-h transfection, cells were lysed with iced IP buffer containing 50 mM Tris-HCl (pH 8.0), 150 mM NaCl, 0.5% sodium deoxycholate, 1% NP-40, a protease inhibitor cocktail, and a phosphatase inhibitor cocktail. The cell lysates were precleared using Protein A/G-agarose beads (Thermo) and next incubated with the indicated antibodies overnight at 4 °C. The immunocomplex was collected and subjected to western blot analyses with the corresponding primary and secondary antibodies. For the ubiquitination assay, cells were co-transfected with HA-ubiquitin constructs together with the plasmids of interest. 48 h after transfection, cells were treated with MG132 (10 μM) for 6 h and collected in iced IP buffer supplemented with 1% sodium dodecyl sulfate (SDS). Pulled-down samples were immunoblotted with the indicated antibodies to observe the polyubiquitinated protein bands under different conditions. For the CHX assay, cells were transiently transfected with the indicated plasmids. After 24-h transfection, cells were incubated with 100 μg/mL CHX dissolved in DMSO and were harvested at the indicated time points, followed by immunoblotting.

### Statistics

Data were presented as mean ± standard deviation (s.d.) as indicated in the figure legends. GraphPad Prism Software Version 7.0 (GraphPad, San Diego, CA) was used to present charts. Data were tested for homogeneity of variance by software GraphPad prior to statistical significance evaluation. All the data points of the bar plots are illustrated. The raw data of dot line plots are shown in other forms such as the raw WB images or quantified in bar plots. Histology, IHC, and IF were performed and analyzed in a double-blinded way. Quantitative image analysis of IHC and IF was performed using ImageJ Version 1.53t analysis software (National Institutes of Health). Statistical significance was determined by unpaired two-tailed Student’s *t* test (two groups) or one-way ANOVA (multiple groups). Differences with *p* < 0.05 were considered statistically significant.

### Reporting summary

Further information on research design is available in the Nature Research Reporting Summary linked to this article.

### Supplementary information

Supplementary Figure Legends

Supplementary Tables

Supplementary Figure 1

Supplementary Figure 2

Supplementary Figure 3

Supplementary Figure 4

Original Data File

Supplementary Figure 5

Reporting Summary

## Data Availability

All data related to this work are available within the article or the Supplementary materials. All data are available from the corresponding authors upon reasonable request.
