# Supplementary material for: Talin-1 inhibits Smurf1-mediated Stat3 degradation to modulate β-cell proliferation and mass in mice
Source: Cell Death Dis. 2023 Oct 31;14(10):709. doi: 10.1038/s41419-023-06235-8 (PMC10616178; doi:10.1038/s41419-023-06235-8)
Supplement: Supplementary file 1 — Supplementary Figure Legends [file 41419_2023_6235_MOESM1_ESM.pdf]

Supplementary Figure Legends

Supplementary Fig. 1 Integrative analysis of pancreatic atlas at single cell resolution. A Dot plot of expression of marker genes in each cell type. **B** Proportion of each cell type in patients of autoantibody (AAB), T1D, and T2D. Wilcoxon rank sum test was performed for statistics. ns, $P > 0.05$, *$P < 0.05$, **$P < 0.01$, ***$P < 0.001$, ****$P < 0.0001$, versus control.

Supplementary Fig. 2 Disrupted cell cycle of pancreatic cells in diabetes diseases. A, B Differential expression of genes involved in the progression from G1 to S phase in pancreatic cells in T1D **(A)** and T2D **(B)**, comparing with healthy control. **C** Cell cycle scores, including S score and G2M score, of b-cells in healthy control, AAB, T1D, and T2D**.** Wilcoxon test was performed for statistics. ns, $P > 0.05$, *$P < 0.05$, **$P < 0.01$, ***$P < 0.001$, ****$P < 0.0001$, versus control.

Supplementary Fig. 3 Expression of Smurf1 in pancreatic sections of mice. A, B IHC staining of pancreatic sections for examing Smurf1 expression. Scale bar, 50 μm. Quantitative data **(B)**.

Supplementary Fig. 4 Effect of haploinsufficiency of *Talin-1* and *Stat3* genes in β-cells on the body weight and Smurf1 expression. A Growth curve. **B** TUNEL staining. **C, D** IHC staining of pancreatic sections for examing Smurf1 expression. Scale bar, 50 μm. Quantitative data **(D)**.

Supplementary Fig. 5 Smurf1 expression in pancreatic sections of HFD-fed or *Talin-1$^{fl/fl}$; Ins1-CreERT* mice. A, B IHC staining of pancreatic sections of HFD-fed mice for examing Smurf1 expression. Scale bar, 50 μm. Quantitative data **(B)**. **C** TUNEL staining. **D, E** IHC staining of pancreatic sections for examing Smurf1 expression. Scale bar, 50 μm. Quantitative data **(E)**.
