# Supplementary material for: Talin-1 inhibits Smurf1-mediated Stat3 degradation to modulate β-cell proliferation and mass in mice
Source: Cell Death Dis. 2023 Oct 31;14(10):709. doi: 10.1038/s41419-023-06235-8 (PMC10616178; doi:10.1038/s41419-023-06235-8)
Supplement: Supplementary file 2 — Supplementary Tables [file 41419_2023_6235_MOESM2_ESM.pdf]

Supplementary Tables

Supplementary Table 1. Mouse primer sequences

Gene	Forward primers (5' to 3')	Reverse primers (3' to 5')
Gapdh	TTTCTTCTTGCCTTGGGAGA	AGTTCCGCACTTCATTCAGG
Talin-1	GAAGTGGCCGAGAGTGTGTC	ATCTTCAGCGAAAGCGCAAC
Stat3	GCGGAGAAGCATTGTGAGTGAG	CCAGACGGTCCAGGCAGATG
Pdx1	TCACCTCCACCACCACCTTCC	CCCAGGCTCGGTTCCATTCG
Mafa	CACCATCACCATCACCACCATCAC	TGACCTCCTCCTTGCTGAAGCC
Ngn3	ACTCAGCAAACAGCGAAGAAGCC	GAGTCAGTGCCCAGATGTAGTTGTG
Nkx6.1	CCACACGGCATCAACGACATCC	GCGGAGGCGGACGAGGAG
Pcna	ATATGCCGAGACCTTAGCCACATTG	TCTCTATGGTTACCGCCTCCTCTTC
Ccnd1	TGGATGCTGGAGGTCTGTGAGG	GCAGGCGGCTCTTCTTCAAGG

Supplementary Table 2. Rat primer sequences

Gene	Forward primers (5' to 3')	Reverse primers (3' to 5')
Gapdh	CCGCATCTTCTTGTGCAGTG	CGATACGGCCAAATCCGTTC
Talin-1	CTACCTGGAGCTGCTGGACC	GGACTCATCTGCCTCCTTGG
Stat3	ACCGGATCGCTGAGGTACAA	TGCCCAATCTTGGCTCTCAA
Pdx1	GGTGCCAGAGTTCAGTGCTAATC	ACTTCCCTGTTCCAGCGTTCC

Supplementary Table 3. Antibody information

Antibodies	Source	Cat no.	Application/Dilution
Talin-1	Proteintech	14168-1-AP	WB (1:1000); IF (1:200)
Insulin	ABclonal	A19066	IHC (1:2000)
Insulin	Cell Signaling Technology	C27C9	IF (1:1000)
Stat3	ABclonal	A19566	WB (1:1000); IHC (1:200)
p-Stat3	ABclonal	AP0705	WB (1:1000)
Actin	ZSGB-BIO	TA-09	WB (1:1000)
Tubulin	ZSGB-BIO	TA-10	WB (1:1000)
Pcna	Servicebio	GB12010	IHC (1:200)
Cyclin D1	ABclonal	A19038	WB (1:1000); IHC (1:200)
Smurf1	Proteintech	55175-1-AP	WB (1:1000); IHC (1:300)
Ubiquitin	ABclonal	A19686	WB (1:1000)
HA	Servicebio	GB12939	WB (1:1000)
