# Supplementary material for: Talin-1 inhibits Smurf1-mediated Stat3 degradation to modulate β-cell proliferation and mass in mice
Source: Cell Death Dis. 2023 Oct 31;14(10):709. doi: 10.1038/s41419-023-06235-8 (PMC10616178; doi:10.1038/s41419-023-06235-8)
Supplement: Supplementary file 9 — Reporting Summary [file 41419_2023_6235_MOESM9_ESM.pdf]

Corresponding Author Name: Huiling Cao
Manuscript Number: CDDIS-23-262(

SPRINGER NATURE

Reporting Summary

Springer Nature wishes to improve the reproducibility of the work that we publish. This checklist is used to ensure good reporting standards and to improve the reproducibility. Please respond completely to all questions relevant to your manuscript. For more information, please read the journal's Guide to Authors.

☑ **Check here to confirm that the following information is available in the Material & Methods section:**

- **The exact sample size (*n*)** for each experimental group/condition, given as a number, not a range
- **A description of the sample collection** allowing the reader to understand whether the samples represent technical or biological replicates (including how many animals, litters, culture, etc.)
- **A statement of how many times the experiment shown was replicated in the laboratory**
- **Definitions of statistical methods and measures**: For small sample sizes (n<5) descriptive statistics are not appropriate, instead plot individual data points
  - Very common tests, such as *t*-test, simple χ^2 tests, Wilcoxon and Mann-Whitney tests, can be unambiguously identified by name only, but more complex techniques should be described in the methods section
  - Are tests one-sided or two-sided?
  - Are there adjustments for multiple comparisons?
  - **Statistical test results**, e.g., ***P* values**
  - Definition of **'center values'** as **median or mean**;
  - Definition of **error bars as s.d. or s.e.m. or c.i.**

Please ensure that the answers to the following questions are reported in the manuscript itself. We encourage you to include a specific subsection in the methods section for statistics, reagents and animal models. Below, provide the page number or section and paragraph number.

Statistics and general methods

	**Reported in section/paragraph or page #**
1. How was the sample size chosen to ensure adequate power to detect a pre-specified effect size? (Give section/paragraph or page #)	The sample size estimate statement was reported in page 25, line 493-494.
For animal studies, include a statement about sample size estimate even if no statistical methods were used.	The sample size estimate statement was reported in page 25, line 493-494.
2. Describe inclusion/exclusion criteria if samples or animals were excluded from the analysis. Were the criteria pre-established? (Give section/paragraph or page #)	The inclusion/exclusion criteria was repoted in page 24-25, line 491-493 and 495-497.
3. If a method of randomization was used to determine how samples/animals were allocated to experimental groups and processed, describe it. (Give section/paragraph or page #)	The randomization statement was reported in page 24-25, line 491-493.
For animal studies, include a statement about randomization even if no randomization was used.	The randomization statement was reported in page 24-25, line 491-493.

4. If the investigator was blinded to the group allocation during the experiment and/or when assessing the outcome, state the extent of blinding. (Give section/paragraph or page #)	The extent of blinding statement was reported in page 31, line 629-630.
For animal studies, include a statement about blinding even if no blinding was done.	The statement about blinding were reported in Page 26, line 526-527 and page 31, line 629-630.
5. For every figure, are statistical tests justified as appropriate?	Yes. Statistical significance was determined by unpaired two-tailed Student ' s t test (two groups) or one-way ANOVA (multiple groups). Differences with $p < 0.05$ were
Do the data meet the assumptions of the tests (e.g., normal distribution)?	Yes. The statement was repoted in page 31, line 626-630.
Is there an estimate of variation within each group of data?	Yes. The statement was repoted in page 31, line 626-630.
Is the variance similar between the groups that are being statistically compared? (Give section/paragraph or page #)	Yes. The statement was repoted in page 31, line 626-630.

Reagents

Reported in section/paragraph or page #

6. Report the source of antibodies (vendor and catalog number)	The source of antibodies was reported in Extended Data Table 3.
7. Identify the source of cell lines and report if they were recently authenticated (e.g., by STR profiling) and tested for mycoplasma contamination	The source of cell lines was repoted in page 25-26, line 509-513.

Animal Models

Reported in section/paragraph or page #

8. Report species, strain, sex and age of animals	Animal models information were repoted in page 24-25, line 477-499
9. For experiments involving live vertebrates, include a statement of compliance with ethical regulations and identify the committee(s) approving the experiments.	The statement of compliance with ethical regulations and identify the committee approving the expreiments was reported in page 25, line 497-499.

10. We recommend consulting the ARRIVE guidelines (*PLoS Biol.* **8**(6), e1000412,2010) to ensure that other relevant aspects of animal studies are adequately reported.

Human subjects

Reported in section/paragraph or page #

11. Identify the committee(s) approving the study protocol.	The identification was reported in page 25, line 504-506.
12. Include a statement confirming that informed consent was obtained from all subjects.	The statement was reported in page 25, line 504-506.
13. For publication of patient photos, include a statement confirming that consent to publish was obtained.	N/A
14. Report the clinical trial registration number (at ClinicalTrials.gov or equivalent).	N/A

15. For phase II and III randomized controlled trials, please refer to the CONSORT statement and submit the CONSORT checklist with your submission.

16. For tumor marker prognostic studies, we recommend that you follow the REMARK reporting guidelines.

Data deposition

Reported in section/paragraph or page #

17. Provide accession codes for deposited data. Data deposition in a public repository is mandatory for:<br>a. Protein, DNA and RNA sequences<br>b. Macromolecular structures<br>c. Crystallographic data for small molecules<br>d. Microarray data	The protein, RNA sequences data were reported in Source Data and Extended Data Tables 1, 2, and 3. The human islets scRNA-seq data can be downed from The Human Pancreas Analysis Program (PANC-DB).

Deposition is strongly recommended for many other datasets for which structured public repositories exist; more details on our data policy are available in the Guide to Authors. We encourage the provision of other source data in supplementary information or in unstructured repositories such as Figshare and Dryad. We encourage publication of Data Descriptors (see Scientific Data) to maximize data reuse.

18. If computer code was used to generate results that are central to the paper's conclusions, include a statement in the Methods section under "**Code availability**" to indicate whether and how the code can be accessed. Include version information as necessary and any restrictions on availability.	N/A
